# Genes, fuel, and fate in lung cancer

**DOI:** 10.1042/BST20250546

**Published:** 2026-10-06

**Authors:** Julia S. Scott, Catrin Lutz, Stefan Prekovic

**Affiliations:** 1 University Medical Center Utrecht, Utrecht, The Netherlands; 2 Division of Tumor Biology and Immunology, The Netherlands Cancer Institute, Amsterdam, The Netherlands; 3 Singapore Eye Research Institute, Singapore, Singapore

**Keywords:** cell fate, epigenetics, metabolism, mutation

## Abstract

Cellular plasticity is a central feature of malignant progression, allowing tumour cells to change identity during tumour evolution, therapeutic adaptation, and metastatic dissemination. This issue is especially acute in lung cancer, where the respiratory epithelium is intrinsically permissive to cell-state change. Genetic lesions, chromatin regulation, and metabolic adaptation are often framed as parallel determinants of lineage plasticity. Emerging evidence instead points to functional coupling across these layers. Oncogenic alterations can weaken lineage fidelity and open alternative trajectories, chromatin regulators can stabilise transitional states and enforce new transcriptional programmes, and metabolic rewiring can supply the biochemical conditions required for these states to emerge and persist. Microenvironmental cues further influence which trajectories are selected and maintained. Here, we discuss the genetic, epigenetic, and metabolic determinants of lineage plasticity in lung cancer, focusing on the points at which they intersect. We propose that plasticity is most usefully understood as a coupled regulatory state, arising from the interaction between oncogenic context, chromatin control, and metabolic support. Defining how these dependencies arise and become functionally coupled within high-plasticity transitional states should help identify points at which lineage switching can be intercepted before alternative lineage programmes are stabilised.

## Introduction

Nature versus nurture: the idea that fate is either predetermined or controllable. On an anthropomorphic scale, it is generally accepted that outcomes and fates are controlled by the complex interplay between both sides. It is therefore puzzling that when considering fate on the level of cellular lineage determination, this debate rages on. Prevailing wisdom suggests that cell fate is either determined entirely by the genetic makeup (nature) or cell intrinsic and extrinsic metabolic factors (nurture); however, given the complex nature of all cellular systems, it seems improbable that these factors act purely in isolation.

Oncogenic transformation is a fitting example of cellular rewiring towards alternate fates, whereby the regulation of lineage determination is hijacked to promote almost all features of aggressive and lethal disease. During tumour progression, cancer cells can undergo dedifferentiation [1]; to acquire therapy resistance, they transdifferentiate into novel subtypes [2]; and finally, during metastasis, tumour cells often undergo an epithelial-to-mesenchymal transition [3]. Generally, these switches are associated with certain mutationally defined cancer subtypes, suggesting that these genetic alterations are the key drivers of plasticity. In parallel, metabolic reprogramming is a core hallmark of cancer [4], and the identification of the Warburg effect, preferential usage of anaerobic glycolysis in conditions of oxygen availability [5], demonstrates that metabolic changes are fundamentally associated with malignant cell-state transformations. Furthermore, metabolic shifts have been linked with developmental lineage transitions [6,7], suggesting that altered cellular metabolism and cell fates are intricately linked.

While many cancer types are capable of lineage switching, understanding this process in lung cancer is of pressing need due to its significant disease burden and lethality on a global scale [8]. Lineage plasticity and cell fate determination are particularly relevant in lung cancer, where the intrinsic plasticity of the respiratory epithelium may make adaptive cell-state transitions especially accessible during malignant progression [9]. Recent evidence points to the emergence of a highly plastic cell state during lung cancer progression [10], which was shown *in situ* to contribute to a lethal phenotype [1]. While this state appears to be concentrated in a certain subset of cells [11,12], it was hypothesised that this state acts as a ‘hub’ for transitions, connecting these plastic cells to many differentiated cell types [1,10]. Despite these advances, the contribution of mutations and metabolism in regulating this hub remains to be determined. Here, we bring together evidence from lung cancer studies that describe either genomic events or metabolic adaptations as drivers of lineage transitions and highlight promising examples that describe an interplay between the two facets.

## Influence of genomic alterations on lung cancer cell fate

Lung cancer is histologically stratified into two major classes, non-small cell lung cancer (NSCLC) and small cell lung cancer (SCLC), with NSCLC further subdivided into adenocarcinoma, squamous cell carcinoma, and large cell carcinoma. Although this taxonomy is rooted in morphology, contemporary classification increasingly incorporates immunohistochemistry and molecular pathology, reflecting the fact that histological subtypes often capture underlying lineage states. These states are maintained by distinct transcriptional circuits and recurrent genomic contexts, including lineage-defining factors such as NKX2-1 and SOX2 and subtype-enriched driver alterations [8,13,14]. This is particularly relevant to plasticity: lineage switching in lung cancer does not occur from a blank slate but from a pre-existing cell state whose developmental, epigenetic, and oncogenic context determines which alternative fates become accessible. Accordingly, genomic alterations matter not simply because they associate with particular histologies, but because they can stabilise lineage identity, lower barriers to transdifferentiation, or bias the direction of state change under therapeutic or microenvironmental pressure.

### Mutations

The identification of oncogenic driver mutations transformed the treatment of NSCLC, particularly in EGFR-mutant disease, where genotype-matched therapies can induce substantial initial responses [15,16]. However, these clinical vulnerabilities also exposed a central feature of lung cancer biology: under therapeutic pressure, some tumours escape pathway inhibition not only through resistance-driving mutations but through lineage plasticity. One of the clearest examples is the switching of EGFR-mutant lung adenocarcinoma into transformed small cell lung cancer (T-SCLC), an aggressive resistance state associated with poor clinical outcomes [17]. In this setting, transformed tumours typically retain the EGFR mutation while losing EGFR dependence, and concurrent loss of *TP53* and *RB1* is repeatedly enriched and appears to create a permissive background for histologic transformation [17,18]. Emerging functional and single-cell models further suggest that this transition is stepwise, with tumour cells passing through a stem-like intermediate state before acquiring a fully neuroendocrine phenotype [19]. EGFR-mutant tumours can also shift towards a squamous identity [20], although this route appears less frequent and less genetically stereotyped than T-SCLC, pointing more strongly to broader transcriptional and epigenetic reprogramming. In this context, alterations in *STK11*, *MTAP*, and *CDKN2A* have been reported, in combination with the up-regulation of c-MYC and SOX2 [21], and mechanistic studies are particularly supportive for *STK11*/LKB1, whose loss can facilitate adenocarcinoma-to-squamous lineage switching through disruption of Polycomb (PRC2)-dependent chromatin control [22].

Additional alterations have been described in EGFR-mutant tumours that undergo small cell transformation, including changes affecting WNT, NOTCH, and PI3K–AKT signalling, together with lesions in chromatin regulators, cell-cycle genes, and DNA-repair pathways [23–27]. At present, however, most of these events remain less consistently recurrent and less functionally resolved than the abovementioned *TP53* and *RB1* loss, making it difficult to distinguish true lineage drivers from broader signatures of tumour evolution under treatment pressure. A similar pattern is seen in ALK-rearranged lung adenocarcinoma, where transformation to T-SCLC has also been reported as a resistance mechanism [14,28–30]. Although the number of well-characterised cases remains limited, available genomic and transcriptomic analyses suggest that developmental reprogramming can coincide with genomic instability in this setting, again pointing to a permissive landscape for lineage conversion under targeted therapy.

Evidence outside therapy-induced transformation is more fragmented but still informative. Loss of the lineage factor NKX2-1 can redirect KRAS-driven lung adenocarcinoma towards a gastric programme through FOXA1- and FOXA2-dependent rewiring, showing that disruption of a lineage-maintaining transcriptional circuit is sufficient to unlock an alternative identity [31]. Intriguingly, other lineage-specifying transcription factors appear to be able to outcompete the original driver of identity, overriding the defining gene expression patterns and inducing hybrid-ID states. For example, in NKX2-1-driven LUAD, high expression of the gastric transcription factor HNF4A results in the adoption of an alternative cell state, whereby HNF4A directly binds and activates its canonical targets, along with redistributing NKX2-1 [32]. In parallel, MYC family members appear to do more than mark aggressive disease states in SCLC: the MYC family broadly can drive temporal subtype evolution and neuroendocrine reprogramming, while L-MYC and c-MYC have been specifically linked to distinct lineage states [33]. These observations place transcription-factor circuitry at the centre of fate control, enabling precise determination of which tumour identities can be established and maintained. This argument is strengthened further by recent work showing that EGFR-mutant lung adenocarcinomas can adopt a basal-shift state during EGFR-tyrosine kinase inhibitor (TKI) resistance in the absence of canonical resistance lesions. In these models, loss of NKX2-1 promoted *de novo TP63* expression, a hybrid alveolar–basal programme, and osimertinib resistance, with CDKN2A/B loss enriched in the basal-shift subgroup [34]. Consistent with this broader principle, USP13 was shown to reinforce club-cell commitment towards squamous carcinoma development through effects on lineage-determining factors, including NKX2-1 and SOX2, in part through c-MYC-associated squamous programmes [34].

Taken together, these studies suggest that genetic alterations influence lung cancer cell fate most strongly when they destabilise lineage fidelity and enable access to alternative transcriptional programmes. In this view, mutations define a permissive landscape for plasticity, but additional regulatory mechanisms are required to stabilise new cellular identities. This makes chromatin regulation a logical next layer through which mutational context is translated into durable lineage change.

### Epigenetic modifications

Epigenetic reprogramming has emerged as one of the clearest mechanistic explanations for lineage plasticity in cancer, with therapy-resistant prostate cancer providing an important precedent for how chromatin-based regulation can redirect cell fate [35,36]. Lung cancer now appears to follow a similar logic. In EGFR-mutant lung adenocarcinomas undergoing neuroendocrine transformation, multiomic analyses have shown that the shift towards an SCLC-like state is accompanied by coordinated changes in DNA methylation, chromatin accessibility, and transcription factor regulatory programmes, and may be driven more by transcriptional and epigenomic reprogramming than by the acquisition of new recurrent mutational events [24]. Consistent with this, small cell transformation can be detected through epigenomic profiling of circulating cell-free DNA, further supporting the view that epigenetic changes are a defining component of the process [37]. This model is strengthened by recent work identifying EHMT2-mediated transcriptional reprogramming as a direct driver of neuroendocrine transformation in NSCLC, reinforcing the idea that specific chromatin regulators can actively enforce a cell identity change [38].

Control of lineage plasticity in lung cancer is strongly linked to altered methylation-dependent chromatin states. A major mechanism through which this occurs is the repression of lineage-defining transcriptional programmes. Among the clearest examples is PRC2, whose catalytic subunit EZH2 supports transcriptional repression programmes linked to plasticity in both NSCLC and SCLC [39]. In SCLC, EZH2 supports tumour progression and neuroendocrine lineage programmes through suppression of the TGF-β–Smad–ASCL1 axis [40], while in NSCLC, EZH2 activity has been implicated in regulating epithelial plasticity and tumour colonisation [41]. In KRAS/LKB1-deficient NSCLC models, PRC2 activity has also been functionally linked to lineage switching, placing Polycomb repression upstream of durable cell-state change [22]. It is important to note that loss of *RB1* has been shown to increase *EZH2* expression in SCLC [42], which may indicate that PRC2-mediated lineage reprogramming does not occur completely independently of genetic effects. Additionally, in prostate cancer, there is evidence to suggest that increased phosphorylation by CDK1 controls EZH2 gain-of-function towards lineage transitions [35], which is relevant as aberrant signalling events such as this are often acquired following genetic mutations. While this mechanism has not been explicitly demonstrated in lung cancer, it is possible that a similar effect would be observed given the other mechanistic similarities related to lineage plasticity between these two cancer types, providing further evidence that EZH2 epigenetic reprogramming in this context may not be devoid of a mutational link. Beyond PRC2-mediated repression, other regulators of histone methylation dynamics are also implicated in lung cancer plasticity. *KMT2D* loss has been directly linked to adeno-to-squamous transition in TKI-resistant lung cancer [43], while the H3K4 demethylases *KDM1A*/LSD1 and *KDM5A* help maintain neuroendocrine differentiation in SCLC by repressing NOTCH signalling and sustaining ASCL1-linked lineage programmes [44–46]. Recent work further shows that LSD1 inhibition can drive neuroendocrine-to-non-neuroendocrine reprogramming through induction of ZFP36L1 [44], reinforcing the idea that histone methylation machinery does not merely mark lineage state but actively controls it. In EGFR-mutant adenocarcinoma-to-T-SCLC transformation, methylation changes linked to differential pathway activity likewise support a model in which lineage conversion is driven by extensive transcriptional and epigenomic rewiring rather than acquisition of new recurrent mutations [24]. Whether these methylation-dependent transitions are directly conditioned by metabolic inputs, including methyl-donor availability, remains unresolved.

Histone acetylation provides an additional layer of chromatin regulation in this setting. Deacetylation of histones, catalysed by HDAC enzymes, promotes chromatin compaction and transcriptional repression [47], and in lung cancer has been linked to the maintenance of plastic, therapy-tolerant states. In EGFR-driven disease, HDAC inhibition has been explored as a strategy to overcome epigenetically mediated resistance and can limit lineage switching in pre-clinical models [48]. In transformed EGFR-mutant models, HDAC inhibitors restored EGFR expression and showed synergy with osimertinib, suggesting that aspects of lineage plasticity may remain epigenetically reversible [48]. This translational logic has already moved into clinical testing, with erlotinib plus entinostat evaluated in a randomised phase II trial and gefitinib plus vorinostat assessed in a phase I EGFR-mutant setting [49,50]. More broadly, HDAC activity also appears to support non-neuroendocrine plasticity, as pan-HDAC inhibition prevented the switch from a proliferative SOX2-driven programme to a more invasive SOX9-driven state in lung cancer cells [51]. Emerging evidence also suggests that histone acetyltransferases (HATs), enzymes that deposit acetyl groups onto histone tails, can drive cell state decisions in SCLC. Specifically, lysine acetyltransferase KAT2A was identified as a key lineage-specifying factor in SCLC using AI models and could be selectively targeted by an orally active protein degrader [52]. In other cancer types, inhibitors of HATs are in phase I clinical trials [53], indicating that this could also represent a promising translational route for lung cancer.

Beyond methylation and acetylation, emerging evidence suggests that additional histone modifications may also contribute to cell-state control. Histone lactylation links metabolic state to transcriptional output, with lactate shown to regulate promoter activity through histone lactylation in NSCLC [54], and histone 4 lysine 12 (H4K12) lactylation implicated in chemotherapy resistance in lung cancer brain metastases [55]. Very recent work has also connected histone 2B (H2B) monoubiquitination to tumour cell plasticity in NSCLC [56], extending the relevance of non-canonical chromatin marks beyond lactate-driven signalling. Related observations in other settings point in the same direction, with histone H4K12 lactylation promoting malignant progression in triple-negative breast cancer [57] and H2B monoubiquitination being required for stem-cell differentiation, suggesting that these marks can influence both malignant plasticity and lineage commitment more broadly [56]. Although these pathways remain less well defined than methylation- and acetylation-based mechanisms, they support the view that epigenetic regulation of cell fate extends beyond the canonical histone modifications discussed above.

These findings point to metabolism as a likely upstream constraint on epigenetic plasticity. Chromatin-modifying enzymes do not operate independently of cellular state but rely on metabolite pools that directly fuel or regulate their activity, including SAM, acetyl-CoA, NAD+, and α-ketoglutarate [58], and the emergence of non-canonical histone modifications also suggests an extended influence of metabolic pathway activity beyond what is currently accepted. At the same time, epigenetic programmes can rewire metabolic gene expression, creating self-reinforcing loops between chromatin state and metabolic adaptation. In lung cancer, this reciprocity raises an important possibility: that metabolic rewiring helps create the epigenetic permissiveness required for lineage switching and may help stabilise transitional states once they emerge.

## Influence of metabolism on lung cancer cell fate and plasticity

During development, transitions from stem-like states to terminal lineages are accompanied by major metabolic rewiring. Glycolytic metabolism is generally enriched in pluripotent states, whereas differentiation is frequently associated with greater mitochondrial respiration and oxidative phosphorylation. Whether these shifts are permissive or instructive remains unresolved, but work in pluripotency and reprogramming supports the view that metabolic rewiring can precede and help enforce cell-fate transitions. In that sense, metabolism is an integral part of the machinery through which cell identity can be remodelled [6,7,59,60].

### The role of cell-intrinsic metabolism

The dysregulation of cellular metabolism in lung cancer compared with adjacent normal tissue is well established [61], but direct evidence that metabolic state actively shapes phenotypic plasticity remains comparatively limited. The clearest support currently comes from neuroendocrine SCLC, where increased expression of PGC-1α enhances mitochondrial capacity and biases tumours towards an ASCL1-expressing lineage state [62]. Consistent with this, recent work shows that neuroendocrine SCLC cells carry unusually high ATP demand and a corresponding dependence on oxidative phosphorylation [63]. These findings argue that metabolic state can do more than support lineage programmes; it can also help bias or stabilise them.

One of the most direct examples in lung cancer of a metabolism-to-epigenome-to-lineage axis is ADORA2A-driven proline metabolism [64]. Activation of ADORA2A rewires proline synthesis and promotes SIRT6/7-mediated histone 3 lysine 27 deacetylation, shifting transcriptional output towards a neuroendocrine programme. Genetic ablation or pharmacological inhibition of ADORA2A suppresses neuroendocrine lung cancer growth and can prevent adenocarcinoma-to-neuroendocrine transition in model systems [64]. Related observations in other tumour types support the broader principle that metabolites can regulate cell fate through chromatin, including lactate-driven dedifferentiation in tumour organoids and epigenetic control of metabolic programmes in liposarcoma plasticity [65,66]. Additionally, metabolism and epigenetics are closely linked in glioma by the enzyme isocitrate dehydrogenase (IDH) that produces the oncometabolite D-2-hydroxyglutarate [67]. IDH mutational status defines two distinct subtypes, whereby IDH-mutant gliomas are less aggressive. Nevertheless, these slow-growing IDH-mutant gliomas will eventually transition to fatal disease. Recently, it was shown that this transition was supported by the expansion of a proliferative neural progenitor-like cell population, underscored by a partial reprogramming of permissive chromatin regulators and alterations in DNA methylation, suggesting an epigenetic underpinning for this initially metabolite-defined cell-state transition [68].

While the aforementioned ADORA2A example stands out as one of the strongest current examples in lung cancer that altered metabolism can actively direct lineage change, not all metabolic links to cell state are equally well resolved. Serine metabolism represents a plausible node connecting metabolic state to cancer progression, but the current evidence is more associative than mechanistic. Phosphoglycerate dehydrogenase (PHGDH), the rate-limiting enzyme in *de novo* serine biosynthesis, defines a metabolic subtype of lung adenocarcinoma associated with increased proliferation and poor prognosis [69], while higher PHGDH expression has also been linked to lymph node metastasis in NSCLC [70]. Phosphoserine phosphatase likewise promotes proliferation, migration, and invasion and is associated with more advanced disease [71].

Metal ion homeostasis provides a further route through which metabolism may influence chromatin state and plasticity. CD44 has been shown to mediate iron uptake, promoting histone demethylation and the expression of mesenchymal lineage programmes [72]. In lung cancer, CD44 has also been linked to migration and invasion through ERK–ZEB1 signalling, suggesting that iron-dependent chromatin regulation may contribute to mesenchymal transitions in NSCLC [73]. More broadly, the growing interest in iron- and copper-dependent cell death pathways, ferroptosis and cuproptosis, reinforces the idea that metal metabolism is tightly coupled to tumour cell state and may expose vulnerabilities in plastic populations [74,75].

### Cell-extrinsic metabolism and cell fate

Tumour metabolic states are shaped not only by cell-intrinsic programmes, but also by the metabolic constraints imposed by the microenvironment. Nutrient availability, oxygen tension, stromal interactions, and endocrine cues can all reshape metabolic flux and, in doing so, influence whether plastic states are maintained, destabilised or selected. Environmental nutrient stress is often buffered by autophagy, the major catabolic system that preserves substrate availability during deprivation [76]. In lung cancer, basal autophagy was associated with a highly proliferative, lowly invasive SOX2-positive state, whereas silencing of the autophagic mediator LC3A promoted transition towards a more invasive phenotype [77]. In parallel, dietary methionine restriction suppresses tumour progression and increases chemotherapy response in KRAS/LKB1-mutant autochthonous NSCLC, indicating that extracellular amino-acid availability can shape the behaviour of metabolically adaptable tumours [78]. At present, however, the direct mechanistic link between methionine availability and lineage switching remains to be fully resolved.

Oxygen availability and reactive oxygen species represent a second major extrinsic constraint on tumour metabolism. In the SOX2-positive lung cancer model described above, the proliferative and invasive states were associated with distinct mitochondrial features, including differences in oxygen consumption, ROS levels, and mitochondrial morphology [77]. Hypoxia has also been linked to dedifferentiation-associated features in lung cancer. Recent work directly demonstrated that hypoxia can promote NSCLC stemness, migration, and invasion by enhancing glycolysis and driving lactylation of SOX9 [79], thereby linking the hypoxic microenvironment to both metabolic rewiring and stem-like cell-state acquisition. Additionally, rewiring of the hypoxia-inducible factors HIF1α and HIF2α was associated with up-regulation of pluripotency factors SOX2 and OCT4 in lung cancer tissue, further linking oxygen deprivation with lineage plasticity in this context [80]. Together, these studies highlight hypoxia as more than a generic stress signal; it is a plausible driver of plasticity through metabolically encoded transcriptional control.

Hormonal cues represent another microenvironmental layer linking cell-extrinsic signals to metabolism, chromatin, and cell fate. In lung cancer, the most direct mechanistic evidence currently comes from glucocorticoid signalling: glucocorticoid receptor activation induces a reversible drug-tolerant dormancy state through chromatin remodelling, enhancer looping, and induction of *CDKN1C* [81]. This may be especially relevant as lung carcinomas themselves can synthesise bioactive glucocorticoids, suggesting that local steroid availability may shape tumour behaviour in addition to systemic endocrine input. Evidence for oestrogen and androgen signalling is more heterogeneous but not negligible. Oestrogen receptor (ER) signalling, particularly ERβ, has been linked to invasive behaviour in NSCLC, including promotion of metastasis through a circ-TMX4/CXCR4 axis [82], while androgen receptor signalling has been associated with tumour-promoting and stemness-related programmes, although direct evidence that it drives lineage switching remains limited [83,84].

Finally, niche-derived signals also provide more than anecdotal support for cell-state regulation in lung cancer. In lung adenocarcinoma, a WNT-producing niche sustains WNT-responsive tumour cells with enhanced tumour-propagating capacity, supporting the idea that stem-like and proliferative states can depend on paracrine niche architecture [85]. This dependency is not fixed: organoid studies show that EGFR–RAS activation can confer WNT independence, whereas loss of NKX2-1 imposes WNT dependency and sensitises tumours to WNT-targeting strategies [86]. Cancer-associated fibroblasts (CAFs) provide a second clear example. In NSCLC, CAFs can drive dedifferentiation and reacquisition of stem-like properties through IGF-II/IGF1R/Nanog signalling [87], and in SCLC, lung fibroblasts can induce neuroendocrine-to-non-neuroendocrine reprogramming through IL-6/JAK2/STAT3/c-MYC/NOTCH signalling, with accompanying chemoresistance [88].

## Intersection between genomic alterations and metabolism in controlling cell fate

Direct evidence that genomic alterations and metabolic rewiring cooperate to control cell fate in lung cancer remains limited. The clearest example currently comes from tumours with concurrent KRAS activation and LKB1 loss. In lung cancer, this mutational combination broadens the histological spectrum, consistent with increased lineage infidelity [89]. At the same time, KRAS/LKB1-mutant tumours acquire distinct metabolic liabilities, including dependence on the hexosamine biosynthesis pathway [90], enhanced serine–glycine–one-carbon metabolism [91], increased metabolic activity under nutrient limitation [92], and sensitivity to bioenergetic stress induced by phenformin [93]. These observations suggest that oncogenic context can establish a metabolic state that does not simply support tumour growth but may also help permit or constrain lineage plasticity. This interpretation is strengthened by work showing that LKB1 loss creates a redox imbalance that can directly modulate adenocarcinoma-to-squamous transdifferentiation [94], linking metabolic state to cell-fate change more explicitly. Recent work further suggests that, in KRAS/LKB1-driven lung cancer, this interface is not purely cell-intrinsic but can be reinforced by the tumour microenvironment. In LKB1-deficient KRAS models, adeno-to-squamous transition has emerged as a drug-relevant high-plasticity route marked by ΔNp63 activation, a KRT6A-positive intermediate state, and reduced sensitivity to KRAS inhibition [95]. In parallel, TET2-dependent activation of a STAT3–CXCL5 axis promotes recruitment of triglyceride-laden neutrophils, which transfer lipids to tumour cells via macropinocytosis and thereby fuel squamous transition [96]. Together, these findings suggest that oncogenic lesions can couple lineage plasticity not only to autonomous metabolic rewiring but also to genotype-conditioned metabolic crosstalk with immune cells, creating a feed-forward niche that helps stabilise fate change.

Evidence from other tumour types supports the plausibility of this model. In KRAS-mutant colorectal cancer, SLC25A22-dependent glutaminolysis promotes succinate accumulation, increased DNA methylation, activation of WNT signalling, and induction of the stem-cell marker LGR5, showing that oncogenic mutation and metabolic rewiring can converge on epigenetic control of differentiation state [97]. Likewise, in SDHB-mutant phaeochromocytomas and paragangliomas, metastatic progression has been linked to coordinated epigenetic and metabolic rewiring, again suggesting that baseline genetic lesions can shape cell-state transitions through downstream metabolic adaptation [98].

## Conclusions and future directions

In cell fate determination, the central issue is how oncogenic context, chromatin regulation, and metabolic adaptation become coupled during state change. In lung cancer, the available evidence supports a view of plasticity as a conditional property of this coupling. Genetic lesions shape which lineage trajectories become accessible by weakening lineage fidelity and rewiring the transcription-factor circuits that maintain identity. Epigenetic regulators influence whether these deviations remain transient or become stabilised. Metabolic adaptation determines whether such transitional states can be entered, sustained, and selected, through effects on redox balance, mitochondrial function, metabolite availability, and exchange with the microenvironment. This framework helps explain why distinct oncogenic settings support distinct repertoires of lineage change and why the same general process of plasticity can produce different cellular outcomes depending on the state of the chromatin and metabolic landscape. Furthermore, this view carries an important conceptual implication. The effect of a genetic lesion cannot be fully understood without the metabolic conditions that support the state it opens, and the effect of a metabolic adaptation depends on the lineage and chromatin context in which it occurs. Plasticity therefore emerges from interdependence across these layers, rather than from parallel contributions that can be considered separately (Figure 1). High-plasticity transitional states are especially relevant in this regard, because they are likely to concentrate the points at which oncogenic signalling, chromatin remodelling, and metabolic support become mutually dependent. These states may therefore provide the clearest setting in which to define causality and, at the same time, the most informative setting in which to search for therapeutic vulnerabilities.

**Figure 1:**
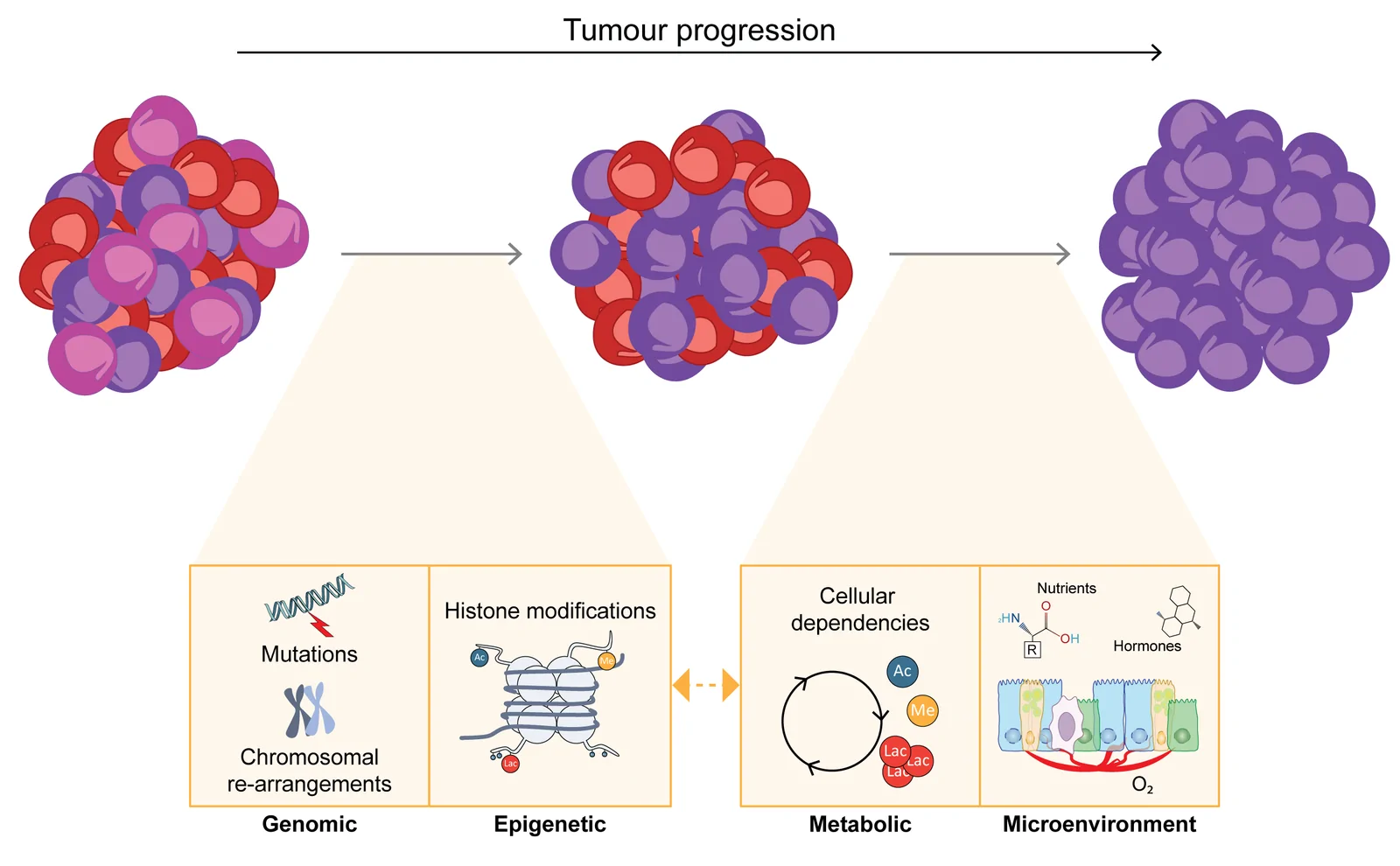
The interconnected network of genetic alterations, epigenetic modifications, cellular metabolism, and microenvironment-derived signals that contribute towards the regulation of lung cancer lineage plasticity Genetic lesions such as mutations and chromosomal rearrangements shape which lineage trajectories become accessible by rewiring transcription-factor activity. Epigenetic regulators influence whether these deviations remain transient or become stabilised and are reliant on metabolic substrates. Cellular intrinsic and extrinsic metabolic adaptations can therefore determine whether such transitional states can be entered, sustained, and selected.

This has direct therapeutic implications. Strategies directed at genotype alone may leave intact the chromatin and metabolic conditions that permit reprogramming, while metabolic intervention without lineage context may produce incomplete or transient effects. A more promising direction is to identify the points at which these dependencies become coupled and therefore vulnerable, including chromatin regulators that rely on specific metabolite states, metabolic adaptations that are selectively required during plastic transitions, and microenvironmental interactions that reinforce fate change in defined oncogenic backgrounds. Similarly, there are opportunities offered by the advancement of spatial-omics techniques that allow for resolution of lineage states, chromatin architecture, and metabolic activity within intact tumour ecosystems. While spatial transcriptomics remains the most advanced [99], spatial metabolomics, proteomics, epigenetic profiling [100,101], and chromatin accessibility [102] techniques are rapidly progressing and will soon be widely implemented. Mechanisms and targets that are identified from simpler model systems and validated within the context of the surrounding tissue architecture and tumour microenvironment are more likely to achieve translational success. Progress will depend on experimental systems that resolve lineage, chromatin, and metabolism within the same framework and capture how these dependencies emerge over time. In lung cancer, the key challenge is therefore to define the coupled molecular conditions that make plasticity possible and to identify where this process can be disrupted before lineage switching becomes fixed.

## Perspectives

- Lineage plasticity is a major determinant of lung cancer progression, contributing to histologic transformation, therapeutic escape, and metastatic dissemination within an epithelium that is intrinsically permissive to cell-state change.
- Current evidence supports a framework in which oncogenic context, chromatin regulation, and metabolic adaptation act together to determine which lineage trajectories become accessible, while microenvironmental cues influence their selection and stabilisation.
- A key priority for the field is to define how these dependencies become coupled within high-plasticity transitional states and to identify the points at which lineage switching remains susceptible to intervention.

## Open Access

Open access for this article is provided via our Subscribe to Open programme, made possible through the support of our subscribing institutions. Learn more here: https://portlandpress.com/pages/open_access_options_and_prices#conditional.

## Abbreviations

CAFs: Cancer-associated fibroblasts
ER: Oestrogen receptor
H2B: Histone 2B
H4K12: Histone 4 lysine 12
HATs: Histone acetyltransferases
IDH: Isocitrate dehydrogenase
NSCLC: Non-small cell lung cancer
PHGDH: Phosphoglycerate dehydrogenase
SCLC: Small cell lung cancer
TKI: Tyrosine kinase inhibitor
T-SCLC: Transformed small cell lung cancer
